# Longitudinal Evolution of Neuroimaging Findings in Fucosidosis: Expanding the Neuroradiologic Spectrum

**DOI:** 10.7759/cureus.112739

**Published:** 2026-07-15

**Authors:** Rahul Nikam, Azam Eghbal, Terence Sanger, Emily Garavatti

**Affiliations:** 1 Radiology, Children's Hospital of Orange County, Orange, USA; 2 Neurology, Children's Hospital of Orange County, Orange, USA

**Keywords:** basal ganglia mineralization, fuca1, fucosidosis, hypomyelination, lysosomal storage disorder

## Abstract

Fucosidosis is an exceptionally rare autosomal recessive lysosomal storage disorder caused by deficiency of α-L-fucosidase due to pathogenic variants in the *FUCA1* gene. Neurologic involvement is prominent, and magnetic resonance imaging (MRI) frequently provides early diagnostic clues through characteristic white matter and deep gray matter abnormalities. We present an 18-year-old female with genetically confirmed fucosidosis and longitudinal neuroimaging spanning 15 years. Initial MRI at 23 months of age demonstrated confluent symmetric supratentorial white matter T2 hyperintensity and subtle medial medullary lamina hyperintensity within the globi pallidi. Follow-up studies demonstrated progressive basal ganglia involvement with evolving T1 and T2 shortening, and susceptibility changes consistent with mineralization extending to the substantia nigra and red nuclei. Detailed longitudinal evolution of neuroimaging findings, particularly progressive deep gray matter mineralization, has been sparsely described in fucosidosis. This case expands the described neuroradiologic spectrum of fucosidosis by highlighting the temporal evolution of white matter and deep gray matter abnormalities, and underscores the value of MRI pattern recognition over time in prompting consideration of rare lysosomal storage disorders and guiding targeted metabolic and genetic evaluation.

## Introduction

Fucosidosis is a rare autosomal recessive lysosomal storage disorder resulting from deficiency of α-L-fucosidase, leading to progressive accumulation of fucose-containing glycoproteins and glycolipids across multiple organ systems [1, 2]. The disorder was first described by Durand et al. [3]. Clinical manifestations are heterogeneous, ranging from rapidly progressive early-onset neurodegeneration to attenuated phenotypes with slower progression and survival into adulthood [1, 2, 4, 5].

Neurologic involvement is a defining feature and frequently leads to neuroimaging evaluation in children presenting with developmental delay, regression, spasticity, and seizures. From a neuroradiologic standpoint, magnetic resonance imaging (MRI) plays a central role in raising diagnostic suspicion. Reported imaging features include symmetric supratentorial white matter abnormalities, deep gray matter signal alterations, particularly involving the globus pallidus, and progressive cerebral and cerebellar atrophy [4, 6]. However, because of the extreme rarity of the disorder and overlap with other leukodystrophies, diagnosis is often delayed. Most published reports describe imaging findings at a single time point, and longitudinal characterization of neuroradiologic evolution remains limited.

## Case presentation

An 18-year-old female initially presented with global developmental delay in the first year of life. She made slow developmental gains and was walking by two years of age. She did not develop spoken language. Spasticity developed around four years of age, with loss of ability for independent ambulation by six years. When she was eight years old, there were concerns for worsening oculomotor function and seizures, which were well controlled with monotherapy with levetiracetam. Metabolic evaluations were negative, including amino acids, mucopolysaccharides, fatty acids, acylcarnitine profile, urine guanidinoacetic acid, urine mucopolysaccharidoses, carbohydrate deficient transferrin, and carnitine profile (Table 1).

**Table 1:** Metabolic Evaluation MECP2: methyl-CpG binding protein 2 gene.

	Result	Reference Range
Amino acid profile	Elevated glycine at 504, recommend perform organic acid analysis. Remainder of panel not significant	127-341
Acylcarnitine profile	Within normal ranges	NA
Carnitine profile	Within normal ranges	NA
Urine organic acids	No abnormalities detected	NA
Glycosaminoglycans, urine	21.4	0-24
Purine and Pyrimidine, urine	Within normal ranges	NA
MECP2	Negative	NA
Fragile X	Negative	NA
Carbohydrate-deficient Transferrin	A-oligo/Di-oligo ratio 0.015, remainder of panel within range	<0.011

Genetic testing for Fragile X and Angelman syndromes was negative, and microarray revealed a microdeletion at 12q23.1 of unclear significance.

**Figure 1:**
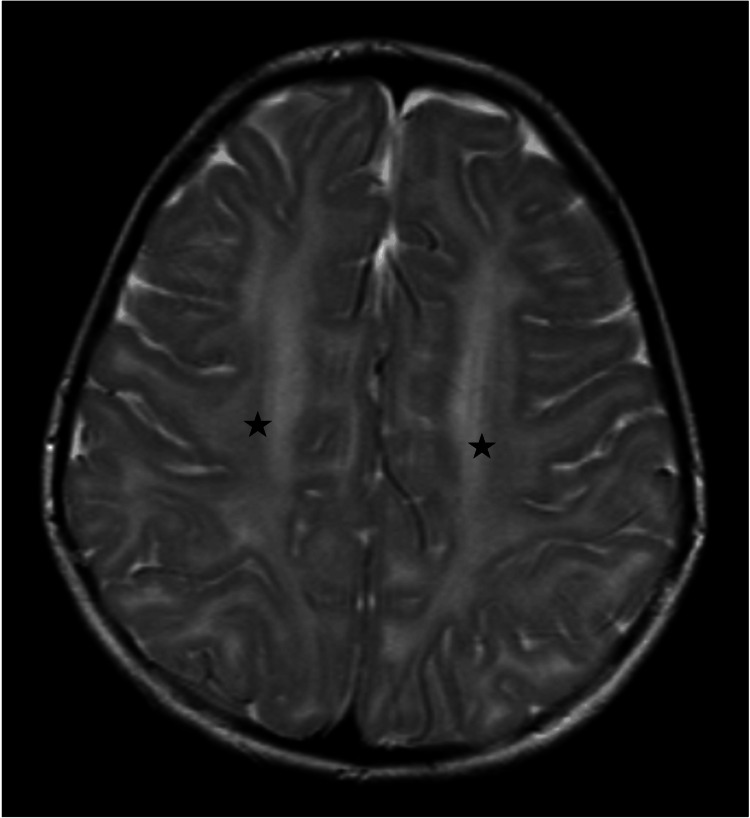
Axial T2 Weighted Imaging Axial T2-weighted image at the level of centrum semiovale obtained at 23 months of age demonstrated extensive, confluent, symmetric T2 hyperintense signal (asterix) involving the periventricular, deep, and subcortical white matter attributed to hypomyelination and metabolite accumulation.

MRI at presentation demonstrated extensive, confluent, symmetric T2 hyperintense signal involving the periventricular, deep, and subcortical supratentorial white matter (Figure 1).

**Figure 2:**
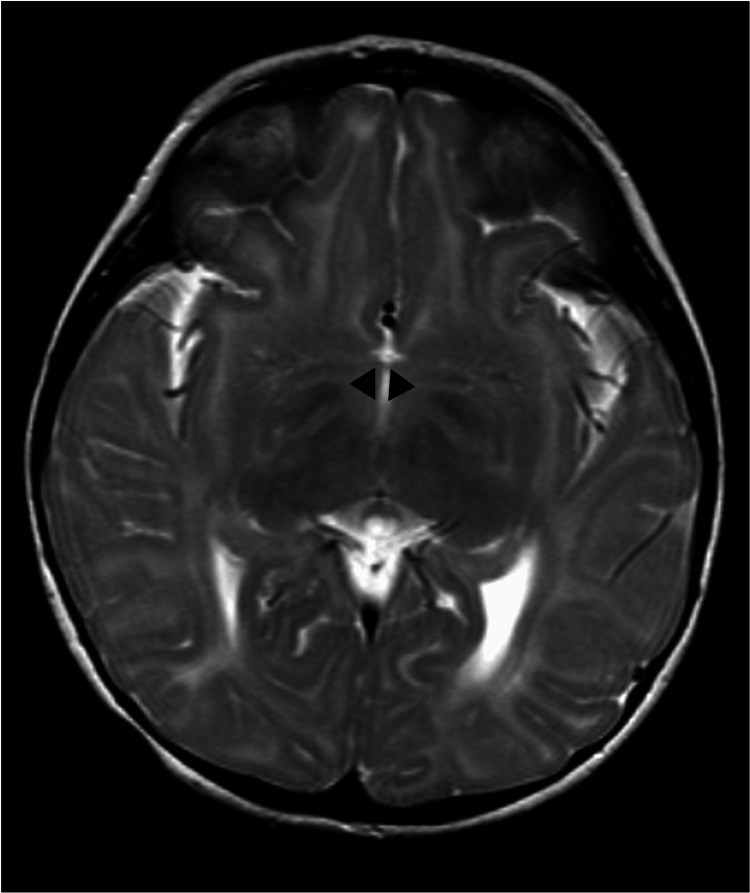
Axial T2 Weighted Imaging Axial T2-weighted image at the level of basal ganglia depicts subtle T2 hyperintense signal within the medial medullary laminae separating the medial and lateral segments of the globi pallidi (arrowheads).

Subtle linear T2 hyperintensity was identified within the medial medullary lamina separating the medial and lateral segments of the globi pallidi, which appeared relatively hypointense (Figure 2).

**Figure 3:**
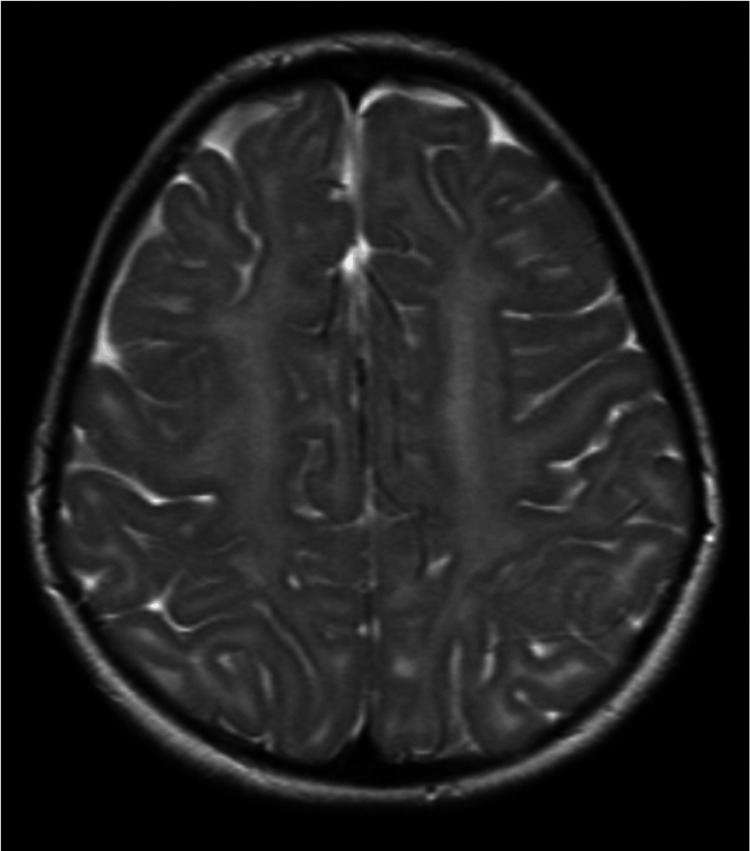
Axial T2 Weighted Imaging Follow-up axial T2-weighted image obtained at 5 years of age demonstrated persistent confluent supratentorial white matter T2 hyperintensity.

Follow-up MRI at five years demonstrated persistent confluent supratentorial white matter T2 hyperintensity (Figure 3).

**Figure 4:**
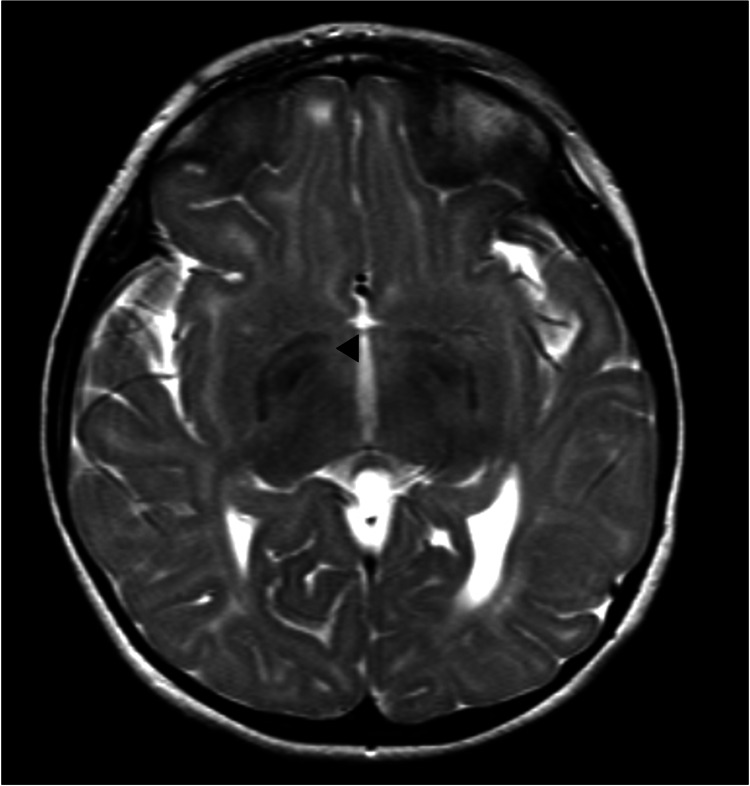
Axial T2 Weighted Imaging The T2 hyperintense signal within the medial medullary lamina appears more well-defined on follow-up MRI flanked by hypointense signal within the medial and lateral segments of the globus pallidus (arrowhead).

The medial medullary lamina hyperintensity became more conspicuous, consistent with previously described pattern-recognition features (Figure 4) [4].

**Figure 5:**
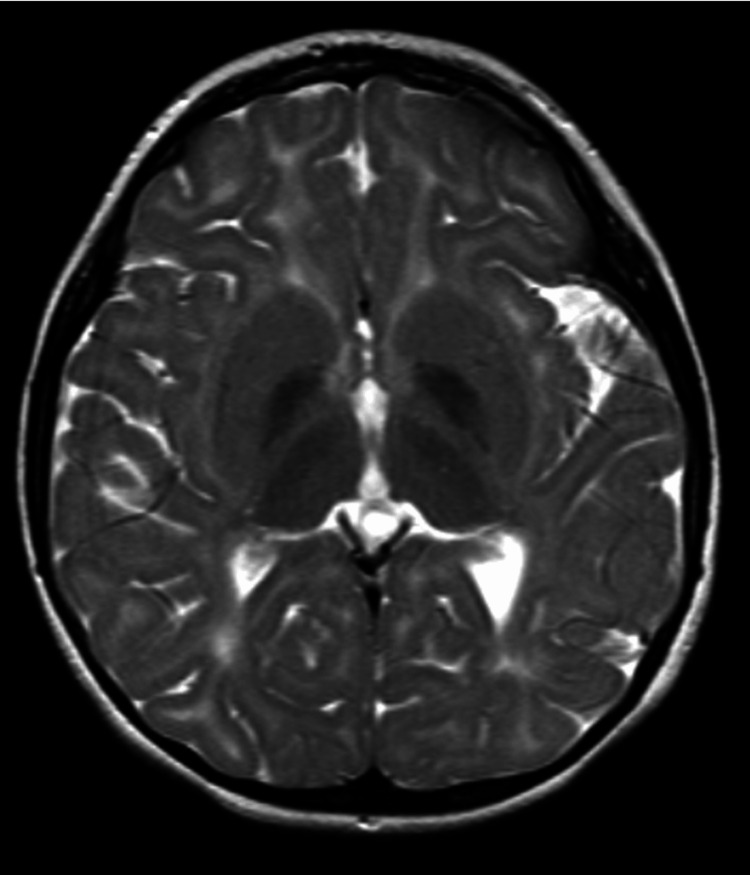
Axial T2 Weighted Imaging Axial T2-weighted image obtained at the level of basal ganglia demonstrated subtle T2 prolongation within the putamina.

In addition, subtle T2 prolongation developed within the bilateral putamina (Figure 5).

**Figure 6:**
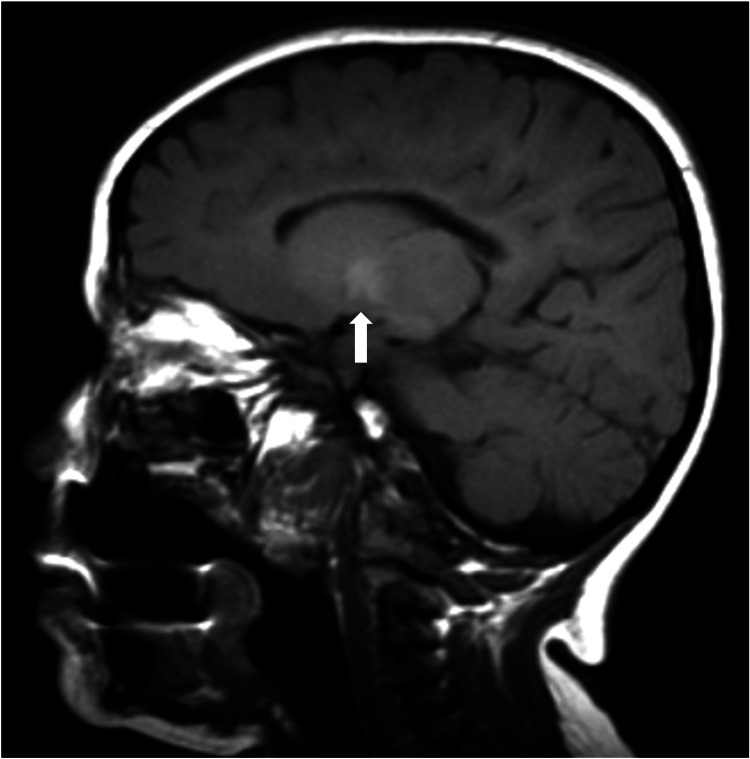
Sagittal T1 Weighted Imaging Sagittal T1-weighted image shows relative hyperintense signal within the globus pallidus with a hypointense streak corresponding to the medial medullary lamina (white arrow).

T1-weighted images demonstrated relative hyperintensity of the globi pallidi with a hypointense streak corresponding to the medial medullary lamina (Figure 6).

**Figure 7:**
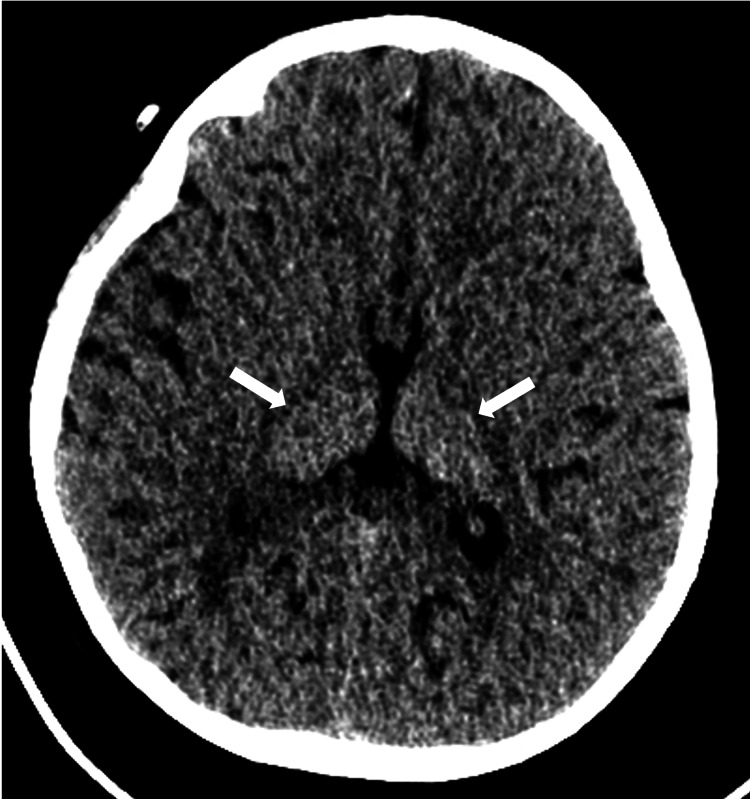
Axial CT Axial non-contrast enhanced CT image in stroke window setting, at the level of basal ganglia obtained at 5 years of age showed relative thalamic hyperattenuation (white arrows).

Non-contrast CT performed at the same time showed relative thalamic hyperattenuation compared with the putamina and caudate nuclei, suggesting evolving mineral deposition (Figure 7).

Due to progressive T2 hypointense signal abnormality in the globi pallidi, a genetic panel for neurodegeneration with brain iron accumulation disorders was sent at 14 years of age, which revealed biallelic pathogenic variants in the *FUCA1 *gene, confirming fucosidosis [7].

**Figure 8:**
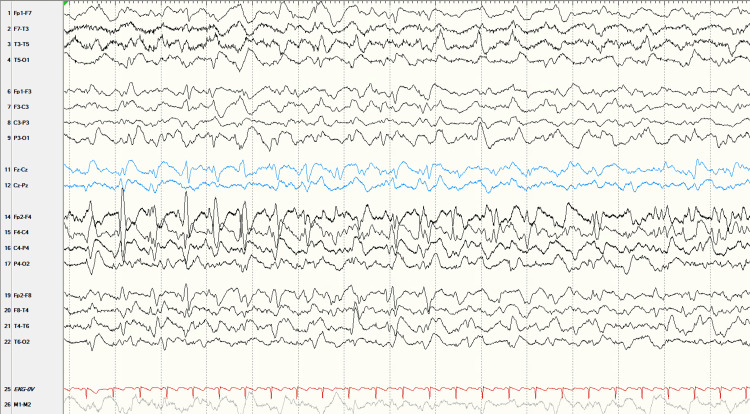
Electroencephalogram (EEG) On EEG, there were diffuse and asymmetric background slowing with absent posterior dominant rhythm, organization and sleep architecture on EEG. There were near-continuous right frontal spike-and-wave discharges at 1 - 2.5 Hz.

The patient’s course was later complicated by bacterial meningitis with a left frontal abscess requiring craniotomy and ventriculoperitoneal shunt placement at 14 years of age. At 18 years, she had two hospital admissions for pneumonia with precipitated seizures and semi-continuous discharges on electroencephalogram (EEG) from the right hemisphere, requiring burst suppression for resolution, and additional anti-seizure medications were added (Figure 8).

**Figure 9:**
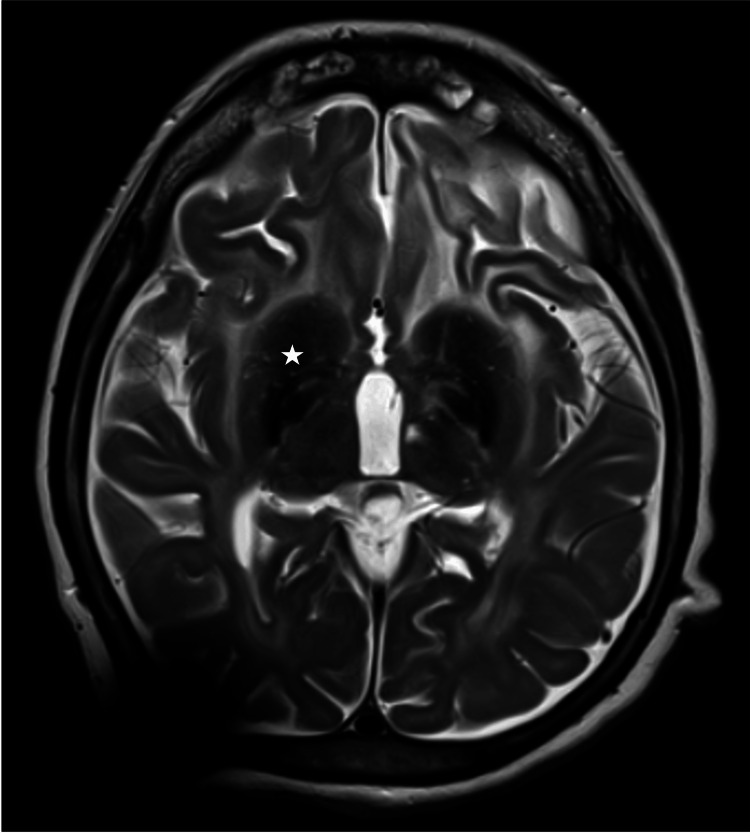
Axial T2 Weighted Imaging Axial T2-weighted image obtained at 18 years of age at the level of the basal ganglia demonstrates diffuse cerebral volume loss, with pronounced T2 hypointense signal within bilateral basal ganglia (asterix), particularly the globi pallidi. There remains relative sparing of the medial medullary laminae. Note, susceptibility artifact with resultant image distortion along the right parieto-occipital region related to ventriculoperitoneal shunt.

**Figure 10:**
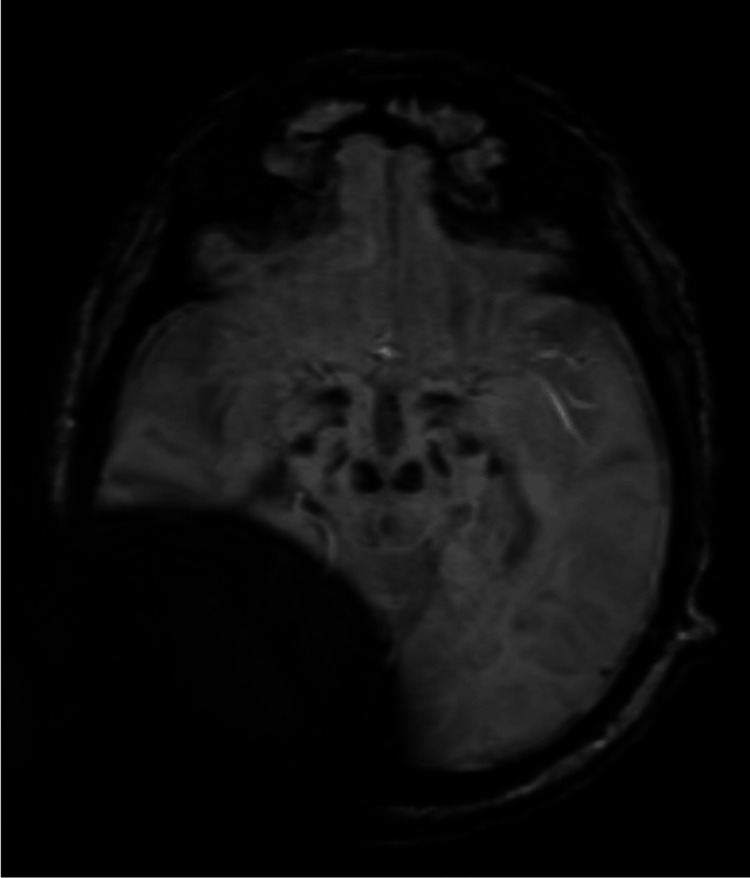
Axial Susceptibility Weighted Imaging Axial susceptibility weighted image the level of mid brain demonstrates pronounced hypointense signal in the substantia nigra and red nuclei. Note, susceptibility artifact along the right parieto-occipital region related to ventriculoperitoneal shunt.

**Figure 11:**
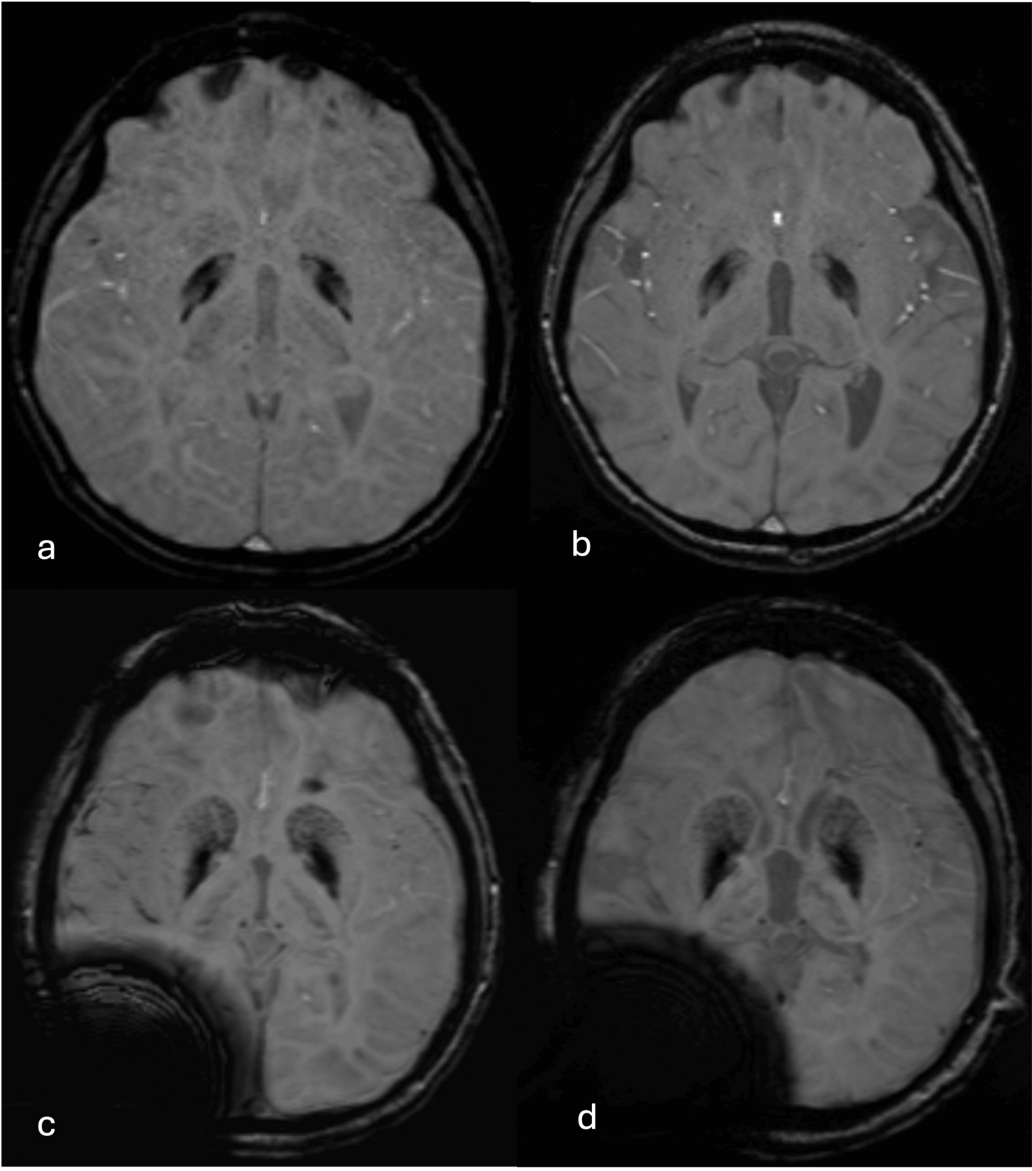
Axial Susceptibility Weighted Imaging Composite susceptibility weighted images obtained at 6 years [a], 8 years [b], 14 years [c], and 18 years [d] of age demonstrate progressively increasing hypointense signal within the basal ganglia, most pronounced in the globi pallidi, consistent with pathologic metabolic mineralization exceeding the expected age-related mineral deposition.

A tracheostomy was placed due to prolonged requirement for respiratory support. MRI at 18 years demonstrated symmetric T1 and T2 shortening with marked hypointense signal on susceptibility-weighted imaging (SWI) involving the bilateral globi pallidi, putamina, substantia nigra, and red nuclei, most pronounced within the globi pallidi (Figures 9, 10, 11) [6]. Notably, there was relative sparing of the medial medullary laminae.

**Figure 12:**
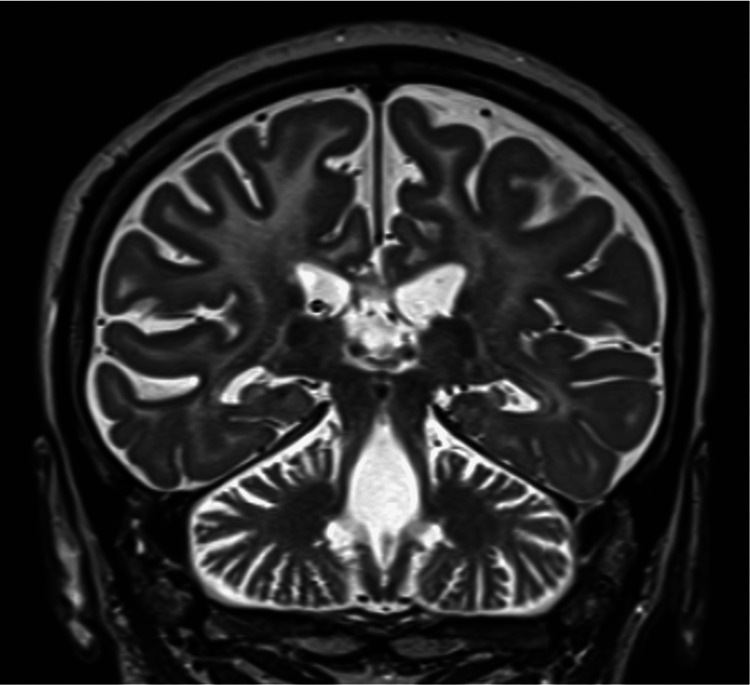
Coronal T2 Weighted Imaging Coronal T2-weighted image obtained at the level of posterior bodies of lateral ventricles demonstrate diffuse marked cerebral and cerebellar volume loss, with persistent extensive, confluent T2 hyperintense signal within the white matter.

Progressive diffuse supra- and infratentorial parenchymal volume loss was evident (Figure 12) [4].

## Discussion

Fucosidosis (OMIM #230000) results from pathogenic variants in the *FUCA1 *gene on chromosome 1p36.11, leading to deficiency of α-L-fucosidase and lysosomal accumulation of fucose-containing substrates [1, 2]. Fewer than 200 cases have been reported worldwide [2]. Two clinical phenotypes are recognized: a severe type I form characterized by rapid neurodegeneration and early mortality, and a milder type II form with slower progression and potential survival into adulthood [1, 2, 5].

Neuroimaging findings reflect the underlying metabolic dysfunction. The hallmark MRI feature is extensive, confluent, symmetric supratentorial white matter T2 hyperintensity, attributed to hypomyelination and metabolite accumulation [4, 6]. These abnormalities often appear early and may precede biochemical confirmation [4]. In our patient, this finding was evident at 23 months and remained a dominant imaging feature throughout follow-up.

Deep gray matter involvement is another important component of the imaging phenotype and ultimately leads to the patient’s diagnosis. Prior reports describe T1 hyperintensity and T2 hypointensity of the globi pallidi, attributed to metabolite deposition and subsequent mineralization [4, 6, 8-10]. In this case, subtle early pallidal signal changes evolved into pronounced T1 and T2 shortening with susceptibility hypointensity involving not only the globi pallidi but also the putamina, substantia nigra, and red nuclei (Figures 9, 10) [6]. The extent and distribution of susceptibility changes exceed the expected age-related mineral deposition and are consistent with pathologic metabolic mineralization. These findings, in our case, prompted gene panel testing for neurodegeneration with brain iron accumulation (NBIA). Due to overlap in clinicoradiologic phenotype, *FUCA1 *is included on the panel for NBIA. Similar patterns have been described in other lysosomal storage disorders such as infantile monosialotetrahexosylyganglioside (GM1) and monosialodihexosylganglioside (GM2) gangliosidosis, supporting a shared mechanism of progressive metabolic neurodegeneration [6]. Neurodegeneration with brain iron accumulation (NBIA) encompasses approximately 10-11 genetically distinct rare disorders characterized by abnormal iron deposition in the basal ganglia and progressive neurological decline. Among these, two subtypes are most prominently associated with hypomyelination or white matter abnormalities: beta-propeller protein-associated neurodegeneration (BPAN) and fatty acid hydroxylase-associated neurodegeneration (FAHN) [7].

Medial medullary lamina hyperintensity has emerged as a distinctive imaging clue in pediatric fucosidosis [4, 9, 11]. In our patient, this feature became more conspicuous over time and also demonstrated relative sparing on late susceptibility imaging despite marked pallidal mineralization. Recognition of this finding may assist in differentiating fucosidosis from other leukodystrophies with basal ganglia involvement. Notably, a similar T2 iso- to hyperintense streaking in the region of medial medullary lamina has been described in some patients with mitochondrial membrane protein-associated neurodegeneration (MPAN), although hypomyelination/white matter signal abnormality is not a described feature [7]. Unlike metachromatic leukodystrophy, which frequently demonstrates a tigroid white matter pattern with relative U-fiber sparing, fucosidosis typically presents with confluent symmetric supratentorial white matter abnormalities accompanied by characteristic pallidal signal alteration [4].

Although MR spectroscopy was not performed in this case, prior reports describe characteristic macromolecule peaks and a 1.2-ppm fructose peak associated with carbohydrate storage products [6, 9]. These findings may further enhance diagnostic specificity.

Reducing diagnostic delay in fucosidosis is clinically consequential, even in the absence of established disease-specific therapy. Earlier recognition can provide diagnostic clarity, avoid prolonged or repetitive investigations, and prompt timely enzymatic and genetic confirmation. A definitive diagnosis also supports anticipatory guidance, multidisciplinary supportive care, and surveillance for neurologic and systemic complications. In addition, because fucosidosis is inherited in an autosomal recessive pattern, early diagnosis has important implications for the family, including parental carrier testing, counseling regarding recurrence risk, evaluation of at-risk relatives, and informed discussion of reproductive options such as prenatal or preimplantation genetic testing when appropriate. Thus, MRI pattern recognition may contribute not only to earlier diagnosis of the affected patient but also to broader genetic counseling and family planning.

The longitudinal imaging evolution over 15 years in this patient provides a unique window into the natural history of neuroradiologic progression in fucosidosis. To our knowledge, detailed serial documentation of progressive susceptibility-weighted mineralization extending beyond the globus pallidus to involve midbrain nuclei has been sparsely described. Serial imaging therefore not only illustrates disease progression but also strengthens diagnostic confidence in rare metabolic disorders.

## Conclusions

Fucosidosis remains a diagnostically challenging lysosomal storage disorder because of its rarity and overlapping imaging features with other leukodystrophies. This case highlights the essential role of MRI in identifying early and evolving abnormalities, including confluent supratentorial white matter involvement, medial medullary lamina hyperintensity, and progressive basal ganglia and midbrain nuclei mineralization. Recognition of these imaging patterns can prompt targeted metabolic and genetic testing and reduce diagnostic delay in this rare disorder.
